# Development and Evaluation of Physiologically Based Pharmacokinetic (PBPK) Models of Metoclopramide Across the Lifespan: Translation from Adults to Infants

**DOI:** 10.3390/ph19091482

**Published:** 2026-09-17

**Authors:** Iqra Shahzad, Ammara Zamir, Muhammad Fawad Rasool, Ali A. Alshamrani, Iltaf Hussain, Faleh Alqahtani

**Affiliations:** 1 Department of Pharmacy Practice, Faculty of Pharmacy, Bahhaudin Zakariya University, Multan 60800, Pakistan; rphiqrashahzad0595@gmail.com (I.S.); ammarazamir20@gmail.com (A.Z.); 2 Department of Pharmacology and Toxicology, College of Pharmacy, King Saud University, Riyadh 11451, Saudi Arabia; aaalshamrani@ksu.edu.sa; 3 Center for Drug Safety and Policy, Xi’an Jiatong University, Xi’an 710061, China; iltafhussain@stu.xjtu.edu.cn

**Keywords:** PBPK model, pharmacokinetics, infants, metoclopramide

## Abstract

**Background**: Physiologically based pharmacokinetic (PBPK) modeling is an established approach used in recent years for estimating drug disposition in challenging clinical scenarios where in vivo studies are difficult to perform. Metoclopramide is a benzamide derivative, widely indicated for its antiemetic and prokinetic actions. This study aims to develop a PBPK model for metoclopramide in infants to predict its systemic exposure in this population. **Methods**: To develop the model, a detailed literature review was conducted, and the required data related to the drug, human physiology, and published clinical studies were retrieved. After that, all information was integrated into the PK-Sim software, and the model was initially developed in adults to create a base; subsequently, it was extrapolated to infants. After successful development, these models were verified visually and numerically using visual predictive checks (VPCs), mean predicted-to-observed ratios (R_pre/obs_), average fold error (AFE), and mean relative deviation (MRD). **Results**: All simulated profiles were consistent with observed data; computed AFE and R_pre/obs_ for key pharmacokinetic (PK) parameters, including area under the concentration–time curve from 0 to t (AUC_0–t_), maximum plasma concentration (C_max_), and clearance (CL), fell within an acceptable two-fold error range. Moreover, the MRD values for all profiles were <2, showing promising agreement between the reported and predicted datasets. **Conclusions**: The current model provides a robust framework to estimate the PK of metoclopramide in infants, which may help with dose individualization in this vulnerable population.

## 1. Introduction

Physiologically based pharmacokinetic (PBPK) modeling is an innovative approach that facilitates the prediction of drug pharmacokinetics (PK) in challenging scenarios, where in vivo clinical trials are difficult to conduct [1]. It aims to provide a mechanistic depiction of the physiological, physicochemical, and biochemical mechanisms controlling drug PK. This is accomplished by structuring the body into anatomically significant compartments, corresponding to distinct organs or tissues, connected by systemic circulation [2]. These PBPK models enable the simulation of drug concentration–time profiles across various pathological and physiological conditions using drug- and system-specific data [2]. They have advanced drug development and regulatory evaluation by providing mechanistic insights into drug release, absorption, and disposition across several populations [3].

The significant lack of comprehensive data on the PK of many drugs in children challenges evidence-based dose optimization; consequently, doses are calculated in this population based on body weight and surface area, neglecting important developmental changes. Age-related critical physiological and biochemical alterations, including ontogenetic variability, organ maturation, and changes in tissue composition, can potentially affect drug disposition; however, computing PK parameters from these changes is complex [4]. Regulatory initiatives to encourage the evaluation and use of drugs in children, led by the United States Food and Drug Administration (US FDA) and the European Medicines Agency (EMA), have increased awareness and concern in this area of research. PBPK modeling can provide a promising framework to bridge the gap between adult and pediatric pharmacology, thus supporting determination of age-specific dose recommendations [5]. These PBPK models enable quantitative anticipation of drug PK in humans by incorporating literature-derived drug-related physicochemical properties, age-specific physiological changes, and population-based information [6]. In the last few decades, numerous studies on the PBPK model in pediatric populations have been published [5,7,8].

Metoclopramide is a derivative of benzamide and belongs to a class of antiemetic and prokinetic agents, indicated for various gastrointestinal tract (GIT) problems [9]. It has a dual mechanism of action: on one side, it antagonizes dopamine D_2_ receptors in the chemoreceptor trigger zone (CTZ) of the brain, contributing to its antiemetic effect, while on the other side, it inhibits peripheral dopamine D_2_ receptors and stimulates serotonin 5-HT_4_ receptors in the GIT, leading to prokinetic activity [10]. The Biopharmaceutics Classification System (BCS) classifies it as a class III drug, depicting its higher solubility and lower permeability [11]. Following oral administration, metoclopramide exhibits rapid absorption, with systemic bioavailability of about 80% [12]. It undergoes N-oxidation and N-deethylation by various Cytochrome P450 (CYP450) enzymes, including CYP2D6, CYP3A4, CYP2C8, and CYP1A2, with CYP2D6 being a major metabolizing enzyme. The major route of elimination for the parent drug and its metabolites is renal excretion; approximately 25% of the drug is excreted unchanged in urine [13].

Gastroesophageal reflux (GER) is a normal and self-limited physiological process in infants, associated with “spitting up” of gastric contents into the esophagus. It is most common among healthy infants aged 1–4 months, with a prevalence of 40–65% [14]. Metoclopramide is commonly prescribed for the management of GER in infants at a dose of 0.1–0.3 mg/kg [15,16]. Although it is not approved by the FDA in infants for any indication, except to facilitate small-bowel intubation for radiological examinations, it has been used off-label in clinical practice to manage GER for years. In addition to GER, it is also prescribed off-label in pediatrics for management of nausea, vomiting, feeding intolerance, and gastroparesis [17,18,19]. Despite its use in infants, safety concerns remain, as the FDA has issued a black-box warning about the tardive dyskinesia associated with overuse of metoclopramide. Moreover, due to the immature metabolic and neurological systems, infants are more vulnerable to the adverse effects of metoclopramide, suggesting dose optimization and careful monitoring [9,16].

The pediatric population is highly heterogeneous, encompassing multiple groups ranging from neonates to adolescence, each with unique anatomical and physiological profiles. According to the International Council for Harmonization E11 (ICH E11), the infant population comprises individuals aged 28 days to 23 months [20]. Rapid developmental variabilities, including organ maturation and altered ontogeny, make this group clinically more important. The level of CYP3A4 rises promptly in infants, reaching approximately 30–40% of adult values by 1 month and 50% at 6–12 months [21]. Moreover, renal excretion changes during infancy, as glomerular filtration reaches adult values by nearly 6 months [22]. This developmental shift creates windows of hypervariability in drug disposition; consequently, adult-derived doses cannot be adequately scaled on a simple body-weight basis [20]. PBPK modeling offers a reliable framework for capturing these rapid developmental dynamics and helps in establishing age-specific dose recommendations [5].

The scarcity of PK data, together with the off-label use of metoclopramide in infants, highlights the need for its evidence-based dose optimization in this population. The developmental changes, including enzyme maturation, organ volumes, and multiple physiological alterations, may affect drug disposition in the pediatric population, increasing the risk of side effects. In this context, PBPK modeling enables the estimation of drug exposure in pediatric populations by integrating drug-specific and age-dependent physiological parameters. Despite clinical recognition, no PBPK model for metoclopramide in infants has yet been developed and published in the literature. Therefore, the current study aims to develop and evaluate a PBPK model for infants to characterize its PK profile. Moreover, the model may help to assess age-related changes in drug exposure, support dosage optimization, and provide a scientific basis for safer and more effective use of metoclopramide.

## 2. Results

### 2.1. Verification of the IV PBPK Model in Adults

**Figure 1:**
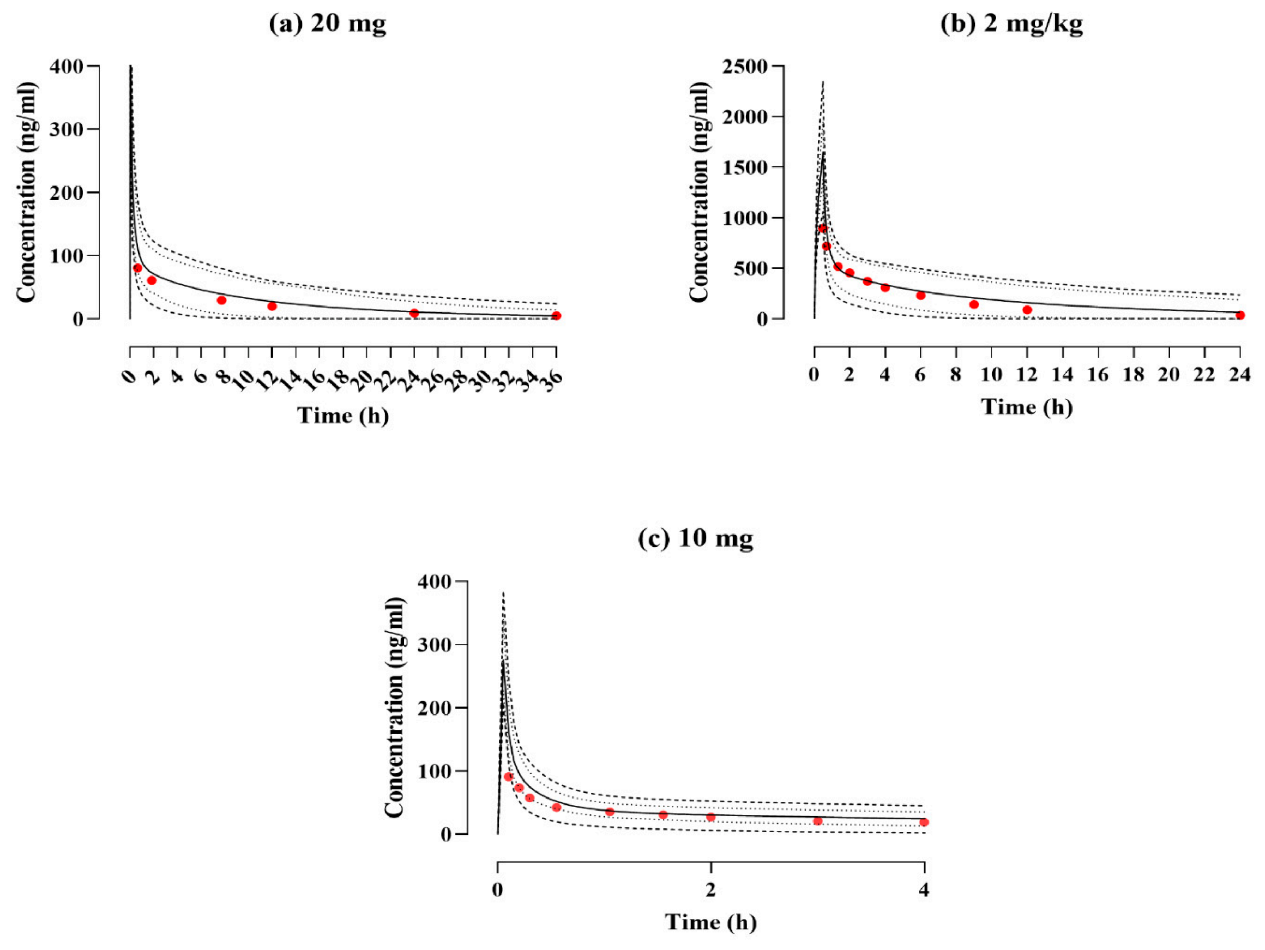
Simulated and observed plasma concentration–time profiles for young adults following IV administration of metoclopramide at doses (**a**) 20 mg, (**b**) 2 mg/kg, and (**c**) 10 mg [23,24,25]. Red dots and solid lines depict reported and predicted data, whereas the 5th–95th centiles and minimum and maximum concentrations are represented by dotted and dashed lines, respectively.

**Figure 2:**
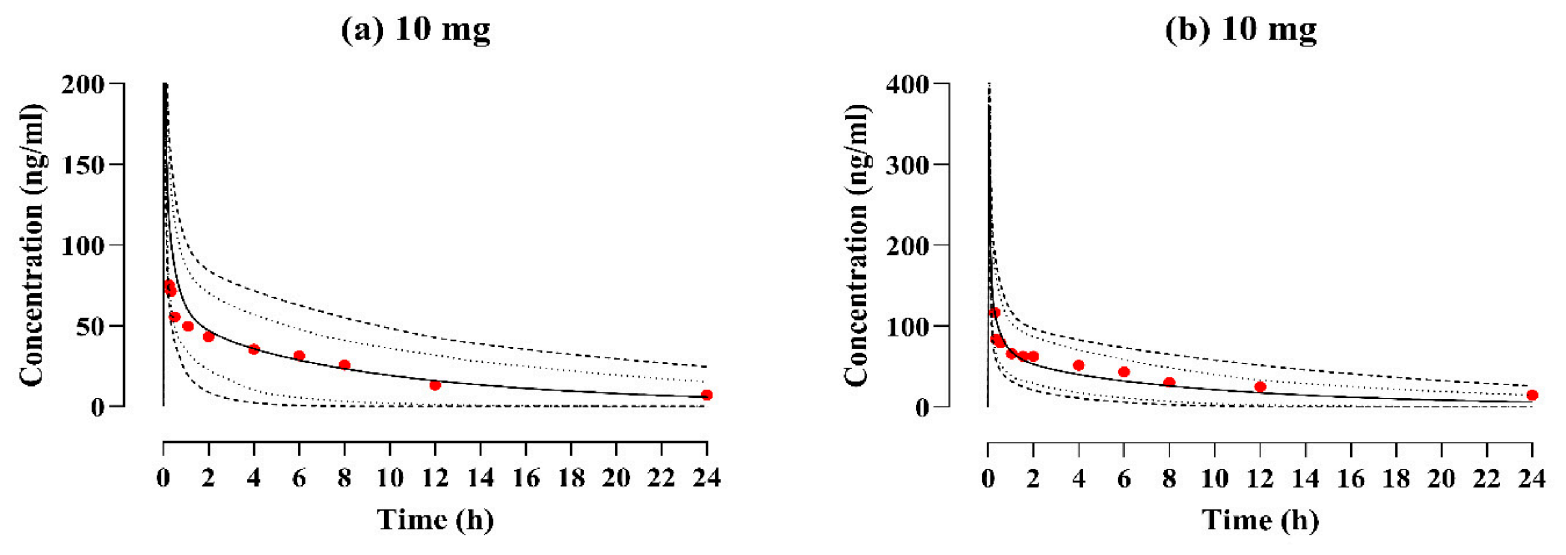
Simulated and observed plasma concentration–time profiles for the elderly following 10 mg IV administration of metoclopramide [26]. Red dots and solid lines depict reported and predicted data, whereas the 5th–95th centiles and minimum and maximum concentrations are represented by dotted and dashed lines, respectively.

The PBPK model-predicted concentration–time profiles following intravenous (IV) administration of metoclopramide in young adults and the elderly were compared with the documented profiles using visual predictive checks (VPCs), and the results were closely aligned with respect to arithmetic mean and 5th–95th percentiles, as illustrated in Figure 1 and Figure 2. The mean predicted-to-observed ratios (R_pre/obs)_ for area under the plasma concentration–time curve from time 0 to t (AUC_0–t_), maximum plasma concentration (C_max_), and clearance (CL) in young and elderly populations were well aligned with an acceptable 2-fold error range. The model’s accuracy was further confirmed, as the average fold error (AFE) for all parameters was within the allowable 0.5–2-fold error range; details are provided in Table 1. The mean relative deviation (MRD) values for all studies related to elderly and young populations were <2, as shown in Table 2.

### 2.2. Verification of the PO PBPK Model in Adults

**Figure 3:**
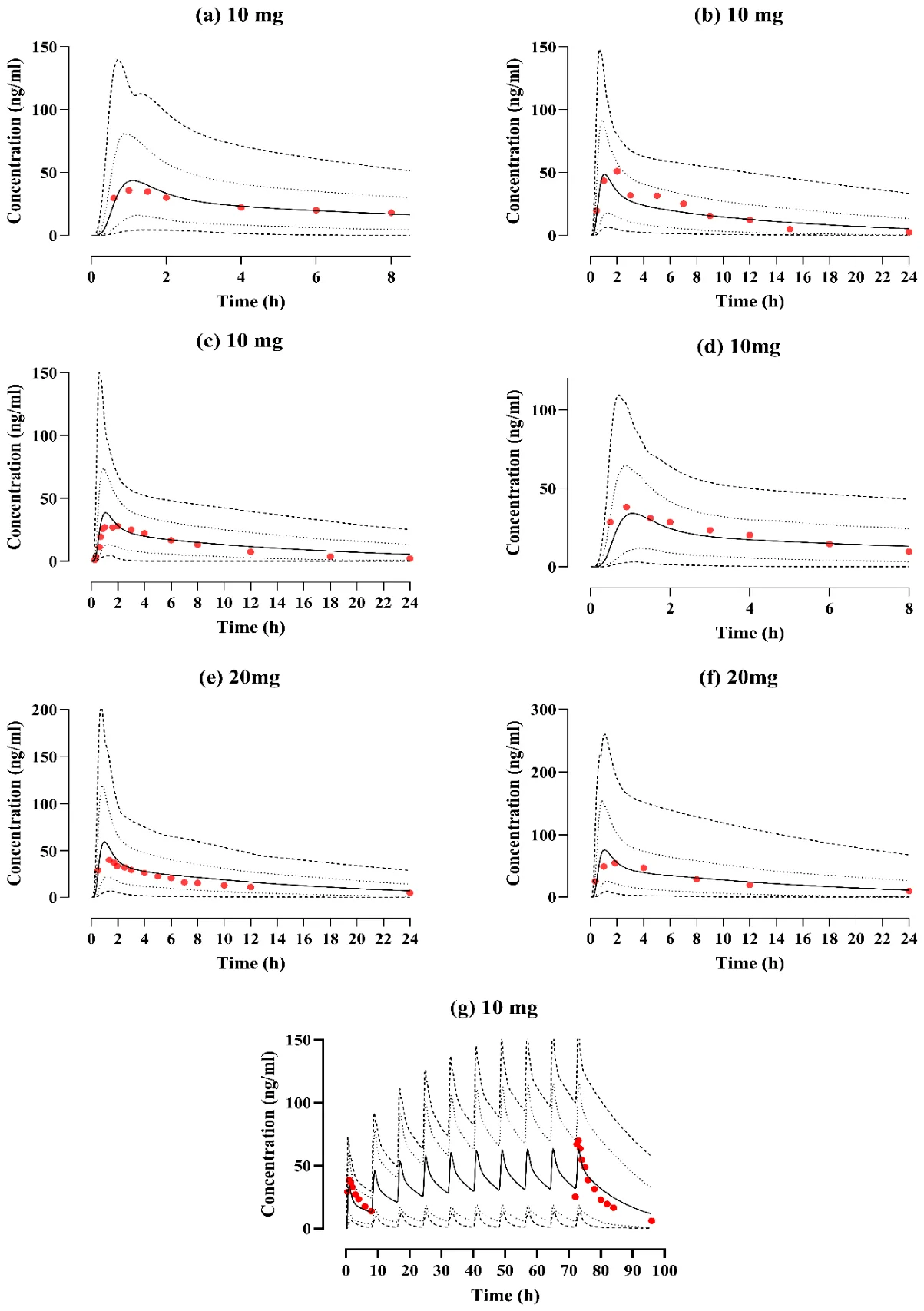
Simulated and observed plasma concentration–time profiles for the young population following PO administration of metoclopramide at doses (**a**–**d**,**g**) 10 mg [26,27,28,29,30] and (**e**,**f**) 20 mg [23,31]. Red dots and solid lines depict reported and predicted data, whereas the 5th–95th centiles and minimum and maximum concentrations are represented by dotted and dashed lines, respectively.

**Figure 4:**
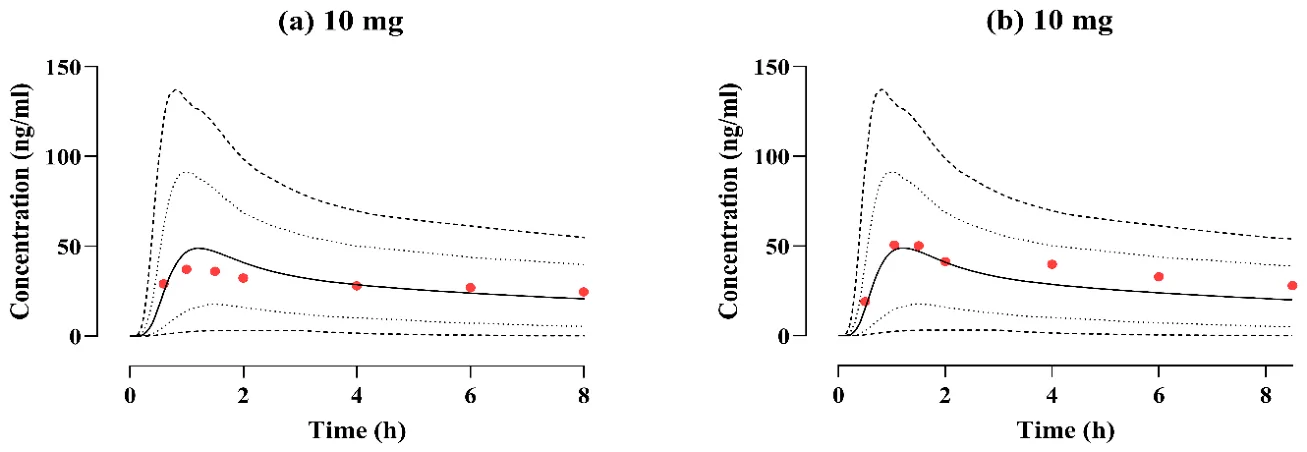
Simulated and observed plasma concentration–time profiles for the elderly population following 10 mg PO administration of metoclopramide [26]. Red dots and solid lines depict reported and predicted data, whereas the 5th–95th centiles and minimum and maximum concentrations are represented by dotted and dashed lines, respectively.

**Figure 5:**
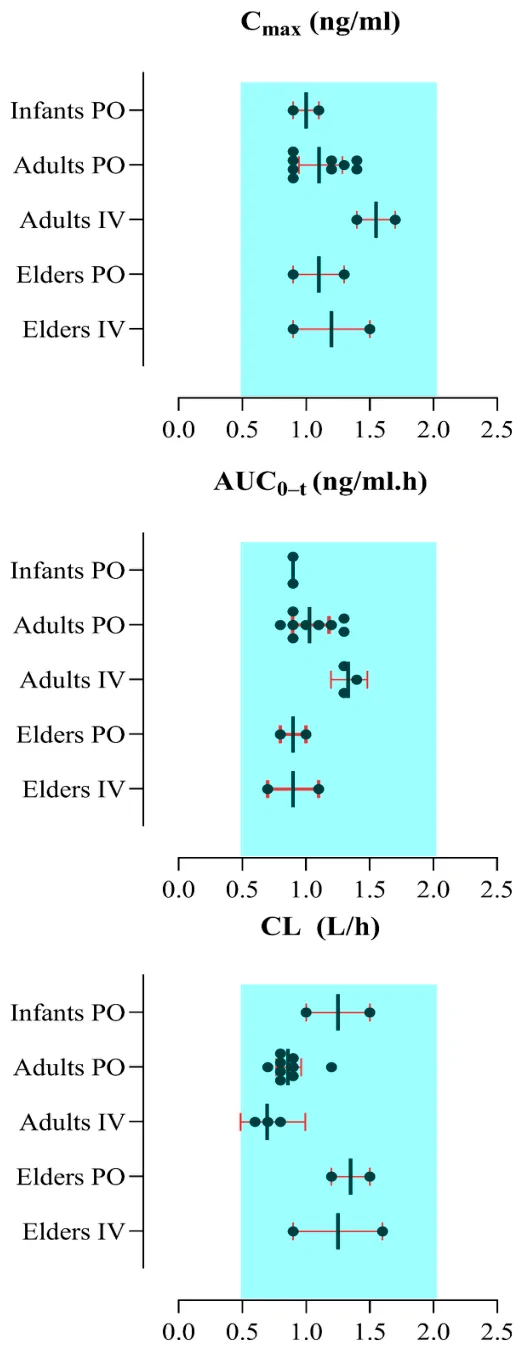
Mean R_pre/obs_ for PK parameters, including C_max_, AUC_0_*_–_*_t_, and CL in adults (young and elderly) after IV and PO administration, and in infants after PO administration of metoclopramide. C_max_: maximum plasma concentration, AUC_0_*_–_*_t_: area under the plasma concentration–time curve from time 0 to t, CL: clearance. Blue shaded area represents acceptable range, i.e., 0.5–2 fold.

The comparison of documented and anticipated profiles following 10–20 mg oral (PO) administration of metoclopramide in both young and older populations is shown in Figure 3 and Figure 4. All profiles were consistent with the arithmetic mean, 5th–95th percentiles, and the minimum and maximum concentrations. The mean R_pre/obs_ for AUC_0–t_, C_max_, and CL were computed and graphically represented in Figure 5. The AFE of AUC_0–t_ for the young and elderly was 1.1 and 0.92, respectively; the details are given in Table 1. The MRD values for all eight and two studies in healthy young and elderly populations, respectively, were within the allowable range of <2 (Table 2).

### 2.3. Verification of the PBPK Model in Infants

**Figure 6:**
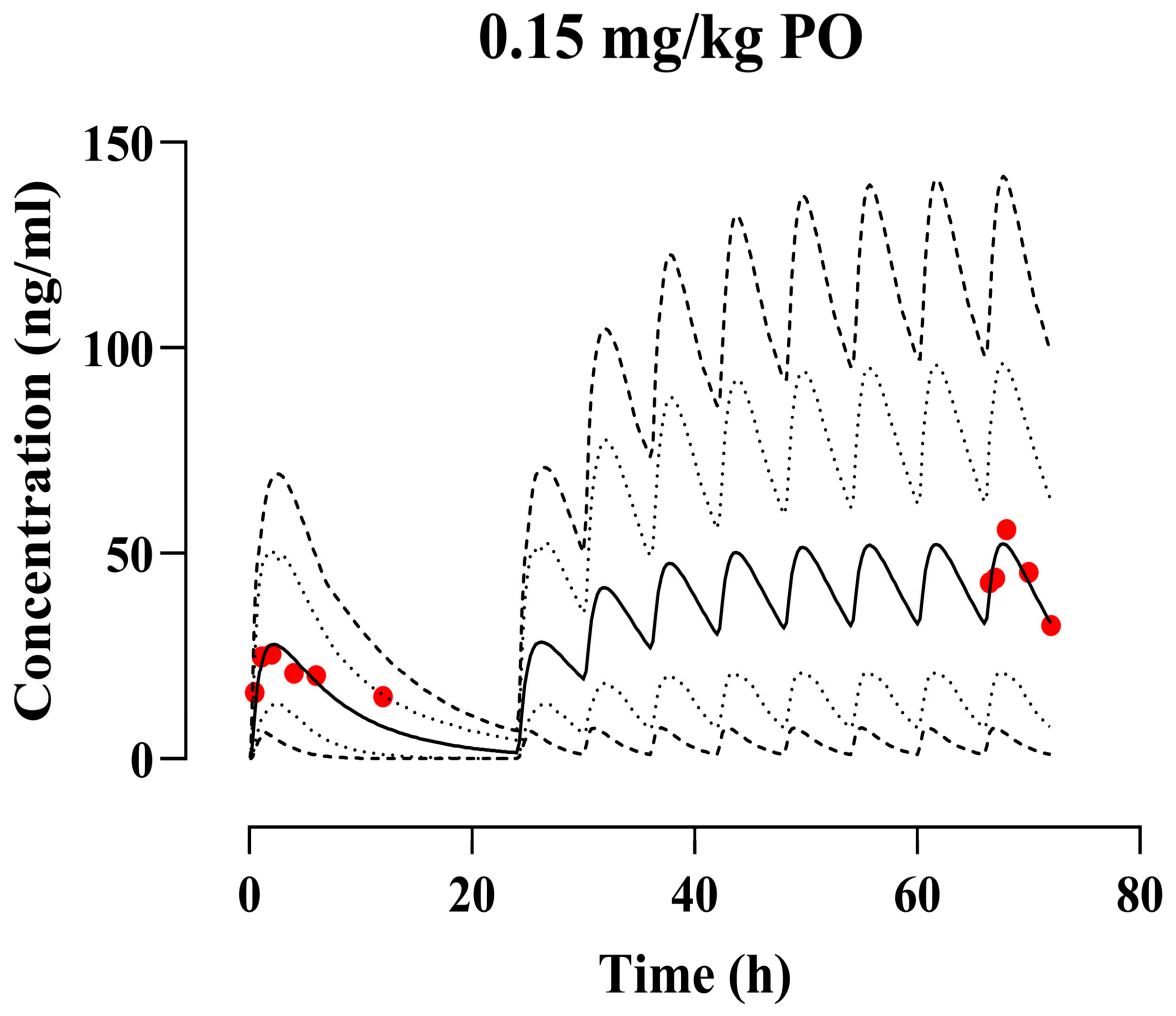
Simulated and observed plasma concentration–time profiles for infants following 0.15 mg/kg PO administration of metoclopramide [15]. Red dots and solid lines depict reported and predicted data, whereas the 5th–95th centiles and minimum and maximum concentrations are represented by dotted and dashed lines, respectively.

Figure 6 compares the clinically recorded and predicted concentrations of metoclopramide in infants following the administration of the first and last doses of 0.15 mg/kg. An acceptable alignment was observed for the arithmetic mean, 5th–95th percentile, and minimum and maximum values of concentrations. The reliability of the PBPK model of metoclopramide in infants was enhanced by computing AFE and R_pre/obs_, presented in Table 1 and Table 2, and the values complied with an acceptable prediction error range, i.e., 0.5–2. The MRD values for the first and last doses were 1.46 and 1.12, respectively.

### 2.4. Anticipation of Metoclopramide Systemic Exposure

**Figure 7:**
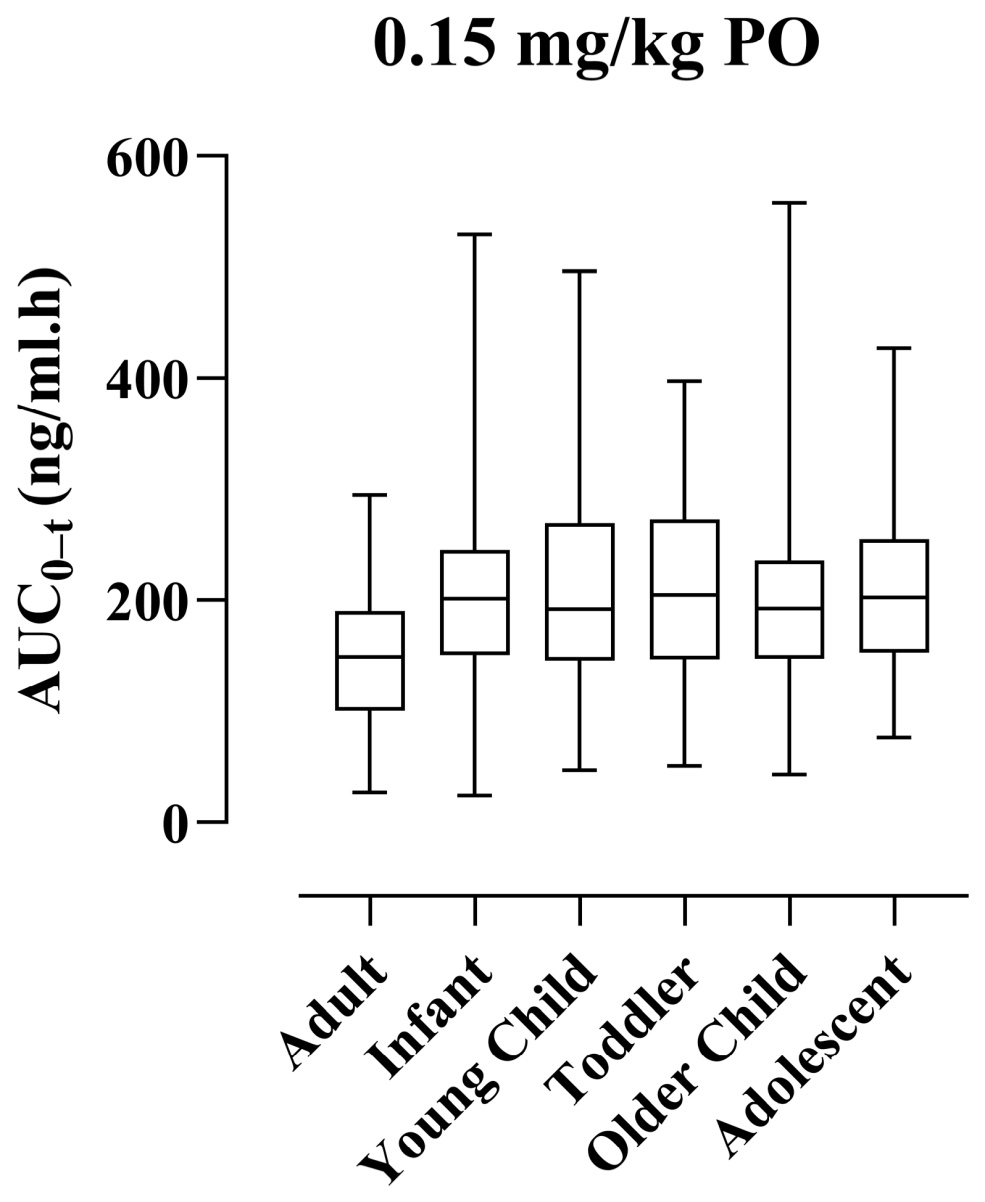
Box-and-whisker plots showing metoclopramide exposure across different age groups.

Box-and-whisker plots were developed to illustrate variability in systemic exposure to metoclopramide across several populations, including infants, young children, toddlers, older children, adolescents, and adults. As no clinical data were available for young children, toddlers, older children, and adolescents, exploratory theoretical simulations were performed across these populations, and the predicted exposures were interpreted as exploratory theoretical estimates rather than verified predictive outputs. For this, virtual populations of 100 individuals were created; subsequently, simulations were performed following a dose of 0.15 mg/kg to show differences in exposure among adults, infants, young children (1–4 years), toddlers (2–3 years), older children (5–11 years), and adolescents (12–18 years), and results are illustrated in Figure 7.

### 2.5. Anticipation of Developmental and Genetic Maturation Differences in CYP2D6

**Figure 8:**
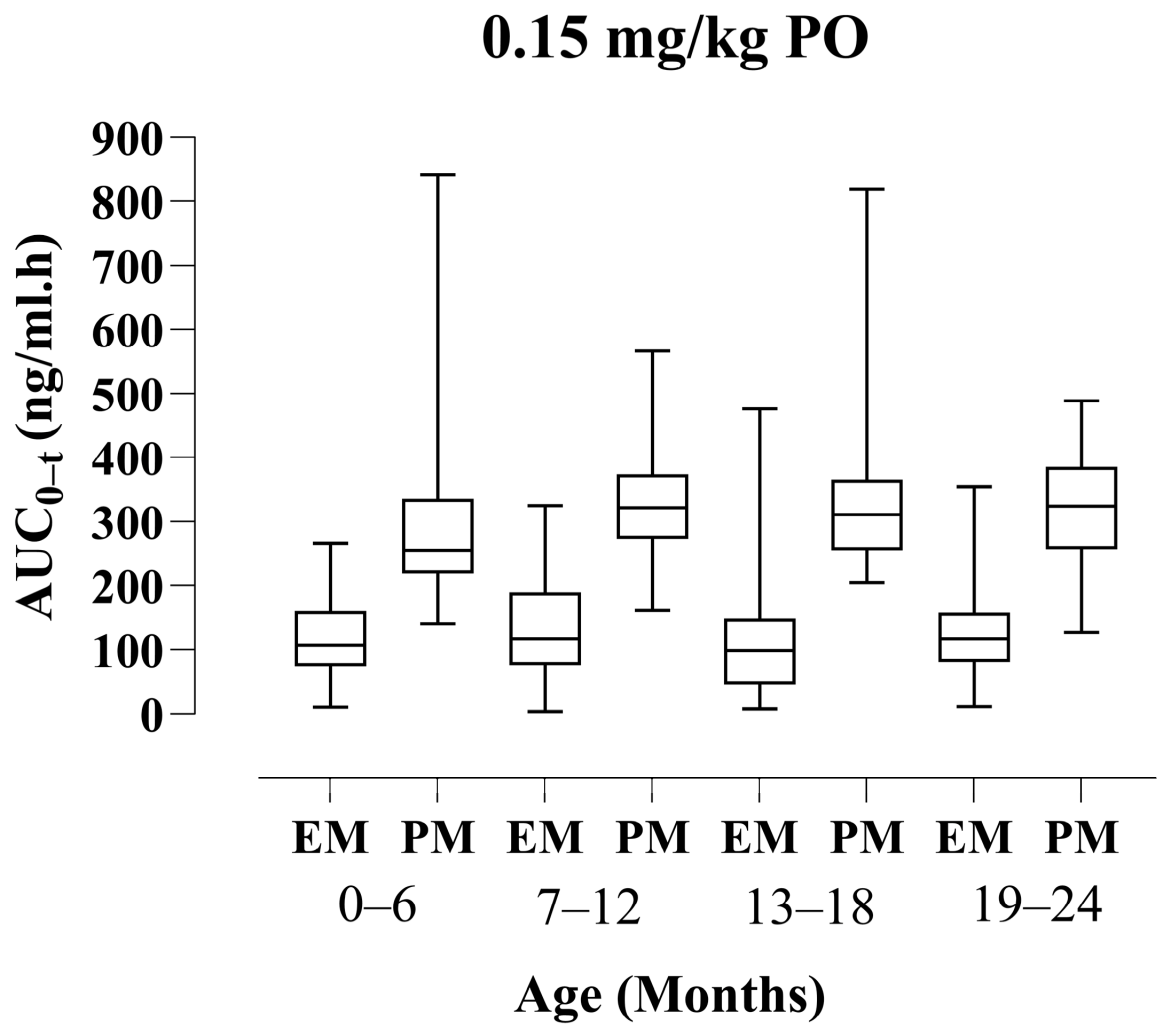
Box-and-whisker plots showing developmental and genetic maturation differences across infancy in CYP2D6 for metoclopramide.

To characterize developmental and genetic maturation of CYP2D6 across infancy, age-stratified analyses were performed for infants aged 0–24 months, an age range approved by the World Health Organization (WHO) [32]. It was initiated by performing exploratory theoretical simulations across multiple age brackets for infants, including 0–6, 7–12, 13–18, and 19–24 months for extensive metabolizers (EM) and poor metabolizers (PM), using Activity Scoring (AS)-based values of turnover number (K_cat_) [33]. Subsequently, box-and-whisker plots were developed to illustrate developmental and genetic maturation differences in CYP2D6 (Figure 8).

## 3. Discussion

The current study has developed a PBPK model for infants, a population with limited clinical data, to characterize the PK of metoclopramide. The model was developed by incorporating PK-Sim’s default age-related changes in organ volumes, cardiac output, blood flow, hematocrit, and plasma protein concentrations, which are known to alter a drug’s ADME. However, the default GET value had overestimated the predicted systemic exposure of metoclopramide in infants. As the literature evidence indicates that GET is higher in infants than in adults, literature-derived values were incorporated to enhance physiological realism and the model’s predictive performance. Several pediatric scintigraphy studies demonstrate that delayed gastric emptying is not consistently present in GER-positive infants, particularly in mild cases, and the GET value in this population is mostly comparable to the normal reference range; therefore, control group data were incorporated into the model [34,35,36]. According to a study, the primary metabolism of metoclopramide is catalyzed by CYP2D6, with additional pathways including oxidation by CYP3A4, CYP2C19, and CYP1A2, as well as conjugation; however, their metabolic parameters (K_m_, V_max_) are not reported in the literature [13]. To address uncharacterized metabolic routes, the PK-Sim-optimized specific hepatic clearance was integrated into the model. Total systemic clearance was not used as an independent input in the model; it represents the net sum of distinct pathways, i.e., $CL_{\mathrm{total}}=CL_{\mathrm{renal}}+CL_{\mathrm{CYP}2D6}+CL_{\mathrm{spec},\;\mathrm{hep}}$ to prevent double-counting metabolic elimination.

Initially, an IV PBPK model of metoclopramide was developed for healthy adults (young and elderly) and visually verified using VPCs. All observed and simulated concentration–time profiles were comparable, indicating agreement across the 5th–95th percentiles, the arithmetic mean, and the minimum and maximum plasma concentrations (Figure 1 and Figure 2). In the young population, the anticipated mean C_max_ after IV administration of metoclopramide was generally higher than the documented value (614.44 ng/mL vs. 355.07 ng/mL), but mean R_pre/obs_ was within the acceptable two-fold error range, as illustrated in Figure 3. Additionally, the predicted AUC_0–t_ showed an increasing trend with dose, i.e., 168.80, 991.08, and 5339.19 ng/mL.h at doses of 10 mg, 20 mg, and 2 mg/kg, respectively. The higher C_max_ after IV administration may be attributed to rapid first-pass tissue distribution and transient uptake to highly perfused organs like the lung, which is the basic characteristic of highly lipophilic drugs including metoclopramide; therefore, in standard well-stirred PBPK models, following IV administration, instantaneous mixing within vascular compartments may lead to overpredicted C_max_ for such drugs [37,38,39]. Additionally, delayed post-dose blood sampling in clinical studies results in lower C_max_ relative to theoretical model estimates [40]. Nevertheless, despite these differences in C_max_, the model has adequately predicted drug exposure across all studies, with C_max_ remaining within the predefined acceptance range. The systematic clearance following IV administration of metoclopramide was underpredicted across studies (AFE=0.70, $R_{\mathrm{pre}/\mathrm{obs}}=0.6-0.8$). This underestimation may result in under-parameterization of minor elimination routes. The computed R_pre/obs_ values for both included studies conducted in elderly subjects were 0.9 and 1.6, falling within the acceptable 0.5–2-fold error range. Models were further assessed by calculating AFE for C_max_, AUC_0–t_, and CL, and all values were consistent with the established two-fold range. The calculated MRD for all profiles of healthy young and elderly individuals was <2, indicating promising agreement between the reported and predicted datasets.

After the IV model, the PO PBPK model for metoclopramide was developed in healthy adults (young and elderly) using PK-Sim software to describe its PK behavior following PO administration. For all profiles (young and elderly), the VPC revealed that reported data were well captured by simulation, as shown in Figure 4 and Figure 5. The model’s predictive ability was further confirmed as the estimated CL for all included studies was in concordance with documented values, detailed in Table 2. Moreover, the AFE values for key PK endpoints, including C_max_, AUC_0–t_, and CL, fell within the two-fold acceptance criteria. The mean R_pre/obs_ ratio for C_max_ among young and elderly populations was 1.1, demonstrating the model’s robustness in reliably predicting metoclopramide PK. The MRD values were <2 across all PO concentration–time profiles (Table 2).

After verification in adults, the model was extrapolated to infants to characterize its predictive performance under age-specific physiological changes. To develop the PBPK model in infants, two literature-derived profiles following the first and last dose of metoclopramide were used. The model’s ability to capture the systemic disposition of metoclopramide despite developmental variability was confirmed as the predicted concentration–time profiles were generally consistent with observed data (Figure 6). The estimated C_max_ for the first and last doses of metoclopramide was in good agreement with the reported values, i.e., 27.72 ng/mL vs. 25.44 ng/mL and 53 ng/mL vs. 55.75 ng/mL, respectively. The estimated CL of metoclopramide in infants after the first dose was 3.68 L/h, which was higher than the observed clinical value of 2.49 L/h. However, the numerical verification via R_pre/obs_ and AFE confirmed the model’s performance, as the results for all included PK parameters (C_max_, AUC_0–t_, and CL) were within the acceptable 0.5–2-fold range. Furthermore, the calculated MRD values for first and last doses of metoclopramide were 1.46 and 1.12, respectively, supporting the model’s predictive reliability. Box-and-whisker plots were developed and illustrated in Figure 7, indicating higher drug exposure in several pediatric populations than in adults. These exposure differences support the need for restricted use to clinically indicated cases with close monitoring of side effects and dose optimization in this vulnerable population.

Box-and-whisker plots were developed to show developmental and genetic polymorphism susceptibility in infants for metoclopramide (Figure 8). The figure describes the physiological interaction between CYP2D6 ontogeny and genetic polymorphism across multiple infant age brackets, including 0–6, 7–12, 13–18, 19–24 months. In early infancy (0–6 months), low CYP2D6 expression reduces the impact of its poor metabolizing capacity on drug exposure, as the difference between AUC_0–t_ was about 2.2-fold between EM and PM. With age, CYP2D6 ontogeny matures, increasing the difference in drug exposure between EM and PM, reaching approximately 3-, 3.2-, and 3.3-fold in the 7–12, 13–18, and 19–24 months groups, respectively.

Model qualification was assessed within a specified Context of Use (CoU), in accordance with regulatory guidance on PBPK modeling and simulation. According to the regulatory “fit-for-purpose” continuum, a model’s qualifying rigor must reflect its clinical risk level [41,42]. Here, high structural confidence was attained by using an established adult model to translate it to infants. Low prediction bias and appropriate precision at the population level are confirmed by the derived infant metrics (AFE, MRD, and Rpre/obs). The model successfully satisfies regulatory requirements for prospective risk assessment, supporting initial model-guided dosing strategies and prioritizing clinical monitoring in the vulnerable infant population, despite parameter uncertainty for individual point estimates resulting from sparse infant datasets.

There is a dearth of observed PK data for metoclopramide in infants because of the ethical constraints associated with pediatric clinical studies. The model’s predictive ability is satisfactory, as it agreed with predefined two-fold criteria of R_pre/obs_ and AFE for available data. Moreover, it is an extrapolation of the adult model, which was evaluated using multiple datasets, reducing the risk of data-driven overfitting. However, limited clinical data restrict the generalizability of this model to infants across different ages, clinical complications, and genetic variability. From a clinical perspective, this infant model cannot be used as a substitute for clinical monitoring to personalize drug therapy. Instead, the model provides a means for refining starting doses to avoid therapeutic overexposure. In clinical practice, the model serves as an exploratory tool to prioritize dose adjustments and design future targeted opportunistic sampling studies in pediatric intensive care settings.

Although the PBPK models of metoclopramide for adults (young and elderly) and infants are well developed as per the two-fold criteria of AFE and R_pre/obs_, this study has several limitations. To integrate reported data for PBPK model verification, plasma concentration–time profiles were assessed point-by-point using GetData Graph Digitizer software; however, this approach may not fully reflect real-time data, which could affect the quality of the findings. Furthermore, the literature on PK studies in infants is limited, which restricts the generalizability of outcomes across different ages and clinical conditions. Extrapolation of the adult PBPK model to infants is also subject to uncertainty related to developmental changes. Despite the incorporation of age-dependent physiological features and enzyme ontogeny, the maturation of drug-metabolizing enzymes, especially CYP2D6, may not be adequately represented by currently available ontogeny functions. Insufficient information on key physiological parameters, including metabolizing enzyme expression, altered GET and GIT fluid volume, and intestinal bile flow, challenges accurate extrapolation of the adult PBPK model in infants. Rapid developmental changes in infants may also introduce uncertainty in predicting drug disposition. Additionally, no infant-specific parameter was optimized, which reduces the risk of overfitting; however, simple extrapolation may increase the chances of propagating the adult model’s uncertainties to infant predictions. Moreover, only two concentration–time profiles were available for infants, which were used to determine gastric emptying time and evaluate the model’s predictive ability. Due to the limited availability of independent infant data, the model verification is considered a preliminary evaluation pending future validation with additional clinical datasets. Notably, no studies have been conducted in other age groups, including toddlers and adolescents. Interindividual variability associated with CYP2D6 polymorphism was not characterized due to scarcity of clinical data. Consequently, more population-based research is needed to understand the variations in PK within the pediatric population. Finally, due to limited availability of GET-related data in infants with GER, baseline values from the control group were used to develop the model; therefore, there is a need to measure GET in symptomatic infants to further refine the predictions.

## 4. Materials and Methods

### 4.1. Literature Exploration and Data Extraction

Specific Medical Subject Headings (MeSH) terms, including “Metoclopramide”, “Pediatrics”, “Infants”, “Pharmacokinetics”, and “Humans”, were used to search Google Scholar and PubMed to retrieve published clinical research reporting concentration–time profiles for metoclopramide. The quality of included studies was assessed by confirming the availability of information on ethical approval, drug administration protocols, and analytical methodologies. In contrast, the exclusion criteria were based on the absence of observed clinical profiles, animal research, and studies involving preterm neonates. A total of 10 studies comprising 15 profiles were extracted from healthy subjects following IV and PO administration of metoclopramide. Among these, 11 profiles were derived from young adults, including 3 following IV and 8 following PO administration, whereas 4 profiles were obtained from older adults, containing 2 after IV and 2 after PO metoclopramide dosing. Moreover, 2 concentration datasets, corresponding to the first and last doses, were obtained from a study of infants with GER. Demographic characteristics and dose-related information obtained from screened studies were used to develop the PBPK model; the details are given in Table 3. An adult model on metoclopramide has previously been developed to investigate the effect of the CYP2D6 polymorphism on drug exposure [43]. The current study extends this work by extrapolating the adult model to infants by incorporating age-based changes.

### 4.2. Computational Platform

In this study, the whole-body PBPK simulation software, PK-Sim version 12.1 (Bayer Technology Services GmbH, Wuppertal, Germany), was employed for PBPK modeling due to its ease of access, user-friendly interface, and advanced features, including disease (chronic kidney disease, chronic liver disease) and ontogeny-based variability [44]. To extract arithmetic values, the reported concentration–time profiles were scanned using GetData Graph Digitizer software, version 2.26 [45]. Moreover, a Microsoft Excel add-in, i.e., PK Solver, was used to compute the required PK parameters from anticipated and documented concentration–time datasets via non-compartmental analysis (NCA) [46].

### 4.3. Modeling Strategy

**Figure 9:**
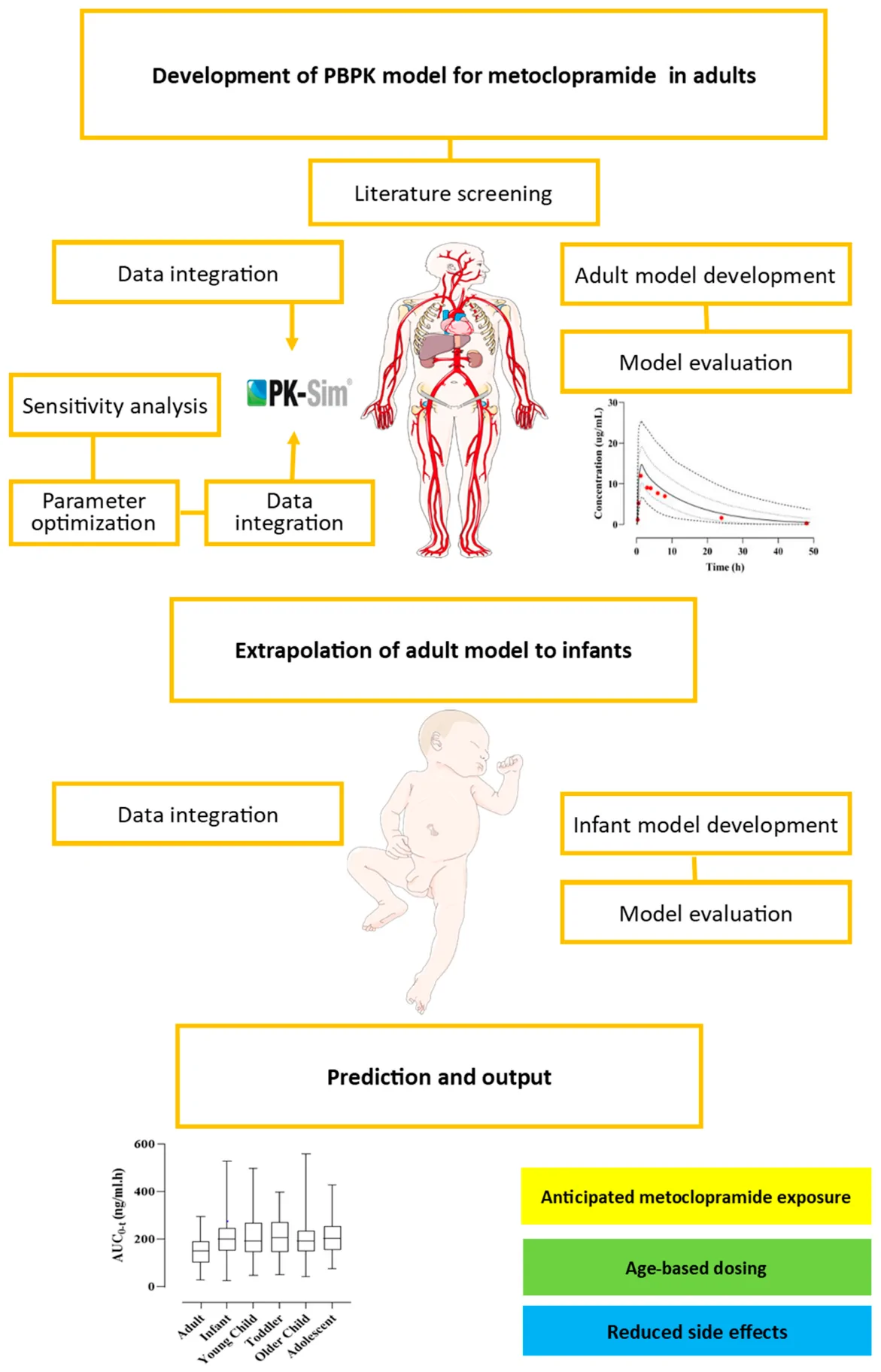
The strategic flowchart for PBPK modeling of metoclopramide. Some units of the diagram were downloaded from Servier Medical Art (SMART), which is licensed under a Creative Commons Attribution 3.0 Unported License https://creativecommons.org/licenses/by/3.0/ (Accessed on 12 March 2026).

Model development was initiated by integrating literature-derived physicochemical, biochemical, physiological, and clinical data on metoclopramide into the PK-Sim software to create building blocks. The preliminary PBPK model was constructed for adults (young and elderly); however, discrepancies between the anticipated and documented data prompted sensitivity analysis to determine the parameters with the greatest impact on model output. Subsequently, the sensitive parameters were optimized either by manual adjustment or by using the parameter identification module of PK-Sim. The optimized parameters were integrated again, and the refined model was evaluated until the simulated data aligned well with the observed data. In the development and verification of the adult PBPK model, one-third (2 IV, 3 PO) and two-thirds (3 IV, 7 PO) profiles were used, respectively, whereas all profiles were employed for final evaluation of the model. After evaluating the model’s predictive performance, it was scaled to infants using PK-Sim, built-in, and literature-reported physiological, anatomical, and ontogenetic data. The schematic framework for model development and evaluation is depicted in Figure 9, and details of the sensitivity analysis are provided in Figure S1 and Table S1.

### 4.4. Model Development in Healthy Adults

This PBPK model was developed using a bottom-up approach following the methodology of published studies [5,47]. The IV model was initially developed to understand baseline PK of metoclopramide to circumvent the complex processes of the PO route. Metoclopramide, having a molecular weight of 299.79 g/mol, predominantly binds with alpha-1-acid glycoprotein due to its basic nature. The literature-parameterized values of solubility, fraction unbound (fu), and dissociation constant (pK_a_) were directly incorporated in the “basic physico-chemistry” section of PK-Sim; in contrast, the lipophilicity (logP) was adjusted within the published range, i.e., 1.8–2.667, to avoid over- or underprediction [48]. At first, the PK-Sim-estimated intestinal permeability was integrated, but overpredicted absorption necessitated refinement of the value from 1 × 10^−5^ to 0.5 × 10^−5^ cm/min. For organ-specific permeability governing tissue distribution, PK-Sim calculated values were retained. To anticipate permeability between the interstitial and cellular spaces and the intracellular-to-plasma partition coefficient, the PK-Sim standard and the Rodgers–Rowland methods were applied. Primary hepatic metabolism of metoclopramide was parameterized using saturable Michaelis–Menten kinetics based on in vitro data of recombinant CYP2D6 obtained from a reference study [13]. The reported value of the Michaelis–Menten constant (K_m_), i.e., 53.3 µM, was integrated unchanged; however, the maximum reaction velocity (V_max_) was optimized from 6.40 to 8.10 pmol/min/pmol to achieve the best model fit. To account for the biotransformation of metoclopramide not attributed to CYP2D6, the optimized specific hepatic clearance, i.e., 1.8 L/h, was incorporated. The renal plasma clearance of metoclopramide is 0.16 L/h/kg, which represents parent drug excretion. For the oral formulation, the Lint 80 approach was applied with a dissolution time of 15 min [11]. The details of input data for PBPK model development are summarized in Table 4.

### 4.5. Scaling of the PBPK Model from Adults to Infants

Following successful development, the adult PBPK model was extrapolated to infants to determine relative alteration in absorption, distribution, metabolism, and excretion (ADME). The built-in PK-Sim^®^ OSP suite database was used to incorporate age-driven changes in body aqueous-lipid content, drug-metabolizing enzyme concentrations, glomerular filtration, anatomical factors, and physiological parameters, i.e., organ volumes, cardiac output, blood flow, hematocrit, and plasma protein concentrations. For the infant cohort, CYP2D6 maturation was parameterized using PK-Sim’s default ontogeny function. The oral solution of metoclopramide was administered to infants; therefore, an instantaneous solution input model of PK-Sim was used to develop an infant model. The anticipated exposure to metoclopramide in infants was overestimated by using PK-Sim’s default gastric emptying time (GET) of 15 min. Due to the unavailability of GET for untreated GER-positive infants, literature-derived scintigraphy data of the control group on gastric emptying half-times (GET ½) were incorporated, with reported values of 63–70 min for infants aged 1–6 months [35]. The GET ½ was converted to the mean GET using Equation (1). The details of the mean GET are provided in Supplementary S1.

$$\mathrm{Mean}\;\mathrm{GET}=\frac{T_{1/2}}{ln(2)}$$

where ln(2) ≈ 0.693.

Parameter optimization was restricted to the adult baseline model to ensure robust extrapolation of the adult PBPK model to the infant population. However, optimization of drug clearance in the adult model may lead to over-attribution to a single pathway. As metoclopramide is mainly metabolized by CYP2D6, integrating age-based ontogeny may provide mechanistic support to translate CYP2D6-mediated metabolism from adults to infants, preserving model generalizability [52].

### 4.6. Evaluation of PBPK Model

The predictive ability of the developed PBPK models was evaluated by employing both numerical and visual methods. The evaluation was initiated by generating populations consisting of 100 individuals using demographic data derived from the respective source publications. The VPC method was used to estimate the alignment between reported and predictive profiles by plotting the arithmetic mean, 5th–95th percentile, and minimum and maximum plasma concentrations. Afterward, all required PK parameters, including C_max_, AUC_0–t_, and CL, were computed via NCA using the Microsoft Excel add-in program, PK Solver. For numerical verification, the model was examined against “R_pre/obs_”, “fold error”, “AFE”, and “MRD” using the following Equations (1)–(4).

(1) $$R=\frac{Predicted\;PK\;parameters}{Observed\;PK\;parameters}$$

(2) $$Fold-error=\frac{Predicted\;values\;of\;parameters}{Observed\;values\;of\;parameters}$$

(3) $$AFE=10\frac{\Sigma log(fold\;error)}{N}$$

(4) $$MRD=10^{x},x=\sqrt{\frac{1}{N}\sum_{i=1}^{N}{\left({log}_{10}C_{Pre,i}-{log}_{10}C_{obs,i}\right)}^{2}}$$

## 5. Conclusions

In conclusion, the IV and PO PBPK models for metoclopramide were successfully developed and evaluated in adults (young and elderly) and were extrapolated to infants. The model demonstrated good predictive ability, as the predicted and observed data for key PK parameters, including C_max_, AUC_0–t_, and CL, were in good agreement and met acceptable two-fold criteria. Despite limited clinical studies and insufficient physiological data, the current model provides a robust framework to characterize the PK of metoclopramide in infants, which may help with dose individualization in this vulnerable population.

## Figures and Tables

**Table 1:** AFE values for metoclopramide adult (young and elderly) and infant PBPK models.

Population	AFE		
	PK Variables		
	C_max_	AUC_0–t_	CL
Young adults (IV)	1.63	1.29	0.7
Young adults (PO)	1.1	1.1	0.84
Elderly (IV)	1.26	0.92	1.3
Elderly (PO)	1.09	0.92	1.35
Infants (PO)	1.01	0.92	1.28

AFE: average fold error, C_max_: maximum plasma concentration, AUC_0–t_: area under concentration–time curve from time 0 to t, CL: clearance.

**Table 2:** R_pre/obs_ ratios and MRD values for metoclopramide PBPK models in adults (young and elderly) and infants.

Sr. No.	Dose (mg)	C_max_ (ng/mL)			AUC_0–t_ (ng/mL.h)			CL (L/h)			MRD
		Obs	Pre	R_pre/obs_	Obs	Pre	R_pre/obs_	Obs	Pre	R_pre/obs_	
Young adults following IV administration											
1	20	80.44	113.49	1.4	771.74	991.08	1.3	23.21	18.91	0.8	1.28
2	10	90.88	161.21	1.7	130.71	168.80	1.3	45.46	26.38	0.6	1.32
3	2 ^a^	895.79	1568.63	1.7	3949.19	5339.19	1.4	31.57	22.61	0.7	1.42
Young adults following PO administration											
1	10	35.79	42.99	1.2	189.07	197.44	1.0	20.0	23.8	1.2	1.13
2	10	51.03	48.40	0.9	370.29	362.50	0.9	25.7	22.8	0.9	1.50
3	10	27.72	38.32	1.4	251.22	335.13	1.3	36.7	25.0	0.7	1.55
7	10	38.52	35.02	0.9	187.75	146.20	0.8	34.7	29.4	0.9	1.53
8	20	55.17	75.86	1.4	600.36	655.79	1.1	26.5	24.1	0.9	1.49
9	10	38.08	32.75	0.9	163.58	146.13	0.9	45.3	38.4	0.8	1.39
10	10	69.74	63.51	0.9	1452.95	1804.03	1.2	6.56	5.04	0.8	1.35
11	20	40.26	53.21	1.3	344.75	442.24	1.3	48.8	37.7	0.8	1.28
Elders following IV administration											
1	10	75.51	117.02	1.5	513.13	560.28	1.1	16.87	15.92	0.9	1.28
2	10	116.74	116.22	0.9	835.55	623.12	0.7	8.65	14.50	1.6	1.38
Elders following PO administration											
1	10	37.14	46.99	1.3	224.93	234.11	1.0	12.8	20.0	1.5	1.21
2	10	50.70	46.88	0.9	287.13	233.62	0.8	16.5	20.0	1.2	1.36
Infants following PO administration											
1	0.15 ^a^	25.44	27.71	1.1	230.84	209.96	0.9	2.49	3.68	1.5	1.46
2	0.15 ^a^	55.75	52.00	0.9	1672.67	1586.04	0.9	0.51	0.52	1.0	1.12

MRD: mean relative deviation, C_max_: maximum plasma concentration, AUC_0–t_: area under concentration–time curve from time 0 to t, CL: clearance, Obs: observed, Pre: predicted, IV: intravenous, PO: per oral, ^a^ mg/kg.

**Table 3:** Patients’ demographic characteristics and dose-related data from retrieved clinical studies used in PBPK modeling of metoclopramide.

Sr. No.	Reference	*N* *	Demographics				Dose-Related Information		
			Age (Years)	Weight (kg)	BMI (Kg/m^2)^	Female Proportion (%)	Dose (mg)	ROA	Dosage Form
1	[26]	6	69–74	-	-	83	10	IV	Bolus
2		6	69–88	-	-	83	10		Bolus
3	[25]	8	23–33	73.5–78.5	-	0	10	IV	Bolus
4	[24]	11	35–65	63–96.5	-	0	2 ^a^	IV	Infusion
5	[23]	8	21–37	52–88	-	63	20	IV	Bolus
6	[26]	6	21–24	-	-	83	10	PO	Tablet
7		6	69–74	-	-	83	10		Tablet
8		6	69–88	-	-	83	10		Tablet
9	[27]	1	-	60	-	100	10	PO	Tablet
10	[28]	41	19–54	49–108	18–30	33	10	PO	Tablet
11	[30]	13	18–39	51.5–79.4	18.26–27.47	0	10	PO	Tablet
12	[29]	13	20.0–50.0	50.8–91.3	18.01–27.44	0	10	PO	Tablet
13	[31]	14	21–29	-	-	0	20	PO	Tablet
14	[23]	8	21–37	52–88	-	63	20	PO	Tablet
15	[15]	6	1–5.5 ^b^	-	-	50	0.15 ^a^	PO	Solution

* Number of individuals participated in study, ^a^ mg/kg, ^b^ months, ROA: route of administration.

**Table 4:** Summarized input data for developing PBPK model of metoclopramide.

Variables	Integrated Data	Source	Rationale
Physicochemical properties			
Molecular weight (g/mol)	299.79 g/mol	PubChem	-
Compound type	Basic	PubChem	-
Plasma protein binding	alpha-1-acid glycoprotein (AAG)	DrugBank	-
Log P (log unit)	2.00	Manually adjusted value	Adjusted within published range
pK_a_	9.33, 9.4,9.27	PubChem	-
Solubility (mg/mL)	5553.2 µg/mL	[49]	-
Reference pH	5.5	[49]	-
Absorption			
Intestinal permeability (cm/min)	0.5 × 10^−5^ cm/min	Manually adjusted value	To avoid overprediction of absorption
Distribution			
Cellular permeabilities model	PK-Sim standard	-	-
Partition coefficients mode	Rodgers and Rowland	-	-
f_u_	0.6	[50]	-
Specific organ permeability (cm/min)	3.96 × 10^−3^ cm/min	PK-Sim estimated	-
Elimination			
Km (µM)			
CYP2D6	53.3	[13]	-
V_max_ (pmol/min/pmol)			
CYP2D6	8.10	Parameter identification	To align observed and predicted data
Total body clearance (L/h/kg)	0.70	[51]	-
Renal clearance (L/h/kg)	0.16	DrugBank	-
Specific hepatic clearance (L/h)	1.8	Parameter identification	To align observed and predicted data

Log P: lipophilicity, PK_a_: dissociation constant, f_u_: fraction unbound, CYP: Cytochrome P450, K_m_: Michaelis–Menten constant, V_max_: maximal rate of enzymatic reaction.

## Data Availability

The original contributions presented in this study are included in the article/Supplementary Materials. Further inquiries can be directed to the corresponding authors.

## Supplementary Materials

The following supporting information can be downloaded at: https://www.mdpi.com/article/10.3390/ph19091482/s1, Figure S1: Sensitivity analysis of fraction unbound, specific intestinal permeability, specific renal clearance, lipophilicity, solubility at reference pH, specific hepatic clearance, and Kcat. Kcat: turnover number; Table S1: Output of PK parameters as a result of sensitivity analysis of fraction unbound, specific intestinal permeability, renal plasma clearance, lipophilicity, and solubility at reference pH, specific hepatic clearance, and Kcat; Supplementary S1: Calculation of Mean Gastric Emptying Time (GET) in Infants.

## Abbreviations

The following abbreviations are used in this manuscript:

PBPK	Physiologically based pharmacokinetic model
PK	Pharmacokinetic
AFE	Average fold error
VPC	Visual predictive check
R_pre/obs_	Predicted to observed ratio
MRD	Mean relative deviation
FDA	Food and Drug Administration
ADME	Absorption, distribution, metabolism, excretion
IVIVE	In vitro–in vivo extrapolation
GIT	Gastrointestinal tract
CTZ	Chemoreceptor trigger zone
USA	United States of America
EMA	European Medicine Agency
BCS	Biopharmaceutics Classification System
GER	Gastroesophageal reflux
AUC_0–∞_	Area under the concentration–time curve from time 0 to infinity
C_max_	Maximum plasma concentration
CL	Clearance
K_m_	Michaelis–Menten constant
V_max_	Maximal rate of enzymatic reaction
k_cat_	Turnover number
MeSH	Medical Subject Headings
IV	Intravenous
PO	Per oral
