# Supplementary material for: Development and Evaluation of Physiologically Based Pharmacokinetic (PBPK) Models of Metoclopramide Across the Lifespan: Translation from Adults to Infants
Source: Pharmaceuticals (Basel). 2026 Sep 17;19(9):1482. doi: 10.3390/ph19091482 (PMC13610140; doi:10.3390/ph19091482)
Supplement: Supplementary file 1 [file pharmaceuticals-19-01482-s001.zip › pharmaceuticals-4506863-supplementary.pdf]

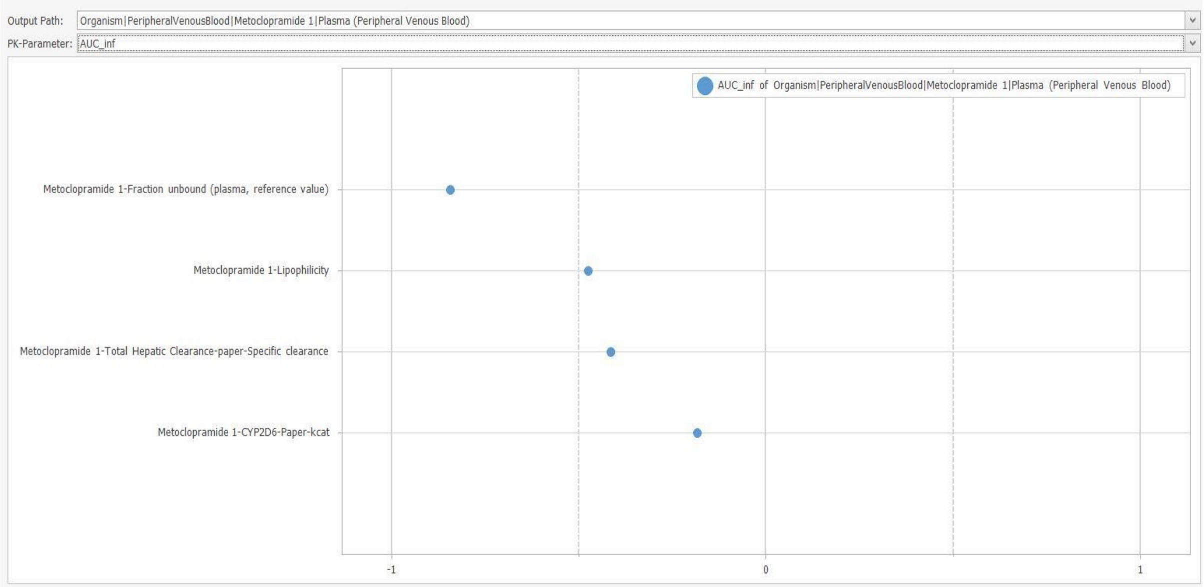

Figure S1. Sensitivity analysis of fraction unbound, specific intestinal permeability, specific renal clearance, lipophilicity, solubility at reference pH, specific hepatic clearance, and $K_{cat.}$ K_{cat}: turnover number

Table S1: Output of PK parameters as a result of sensitivity analysis of fraction unbound, specific intestinal permeability, renal plasma clearance, lipophilicity, and solubility at reference pH, specific hepatic clearance, and K_{cat}

PK Parameter	K_{cat}	F_u	Specific renal clearance	Lipophilicity	Solubility	Specific intestinal permeability	Specific hepatic clearance
% $AUC_{tlast-\infty}$	−0.85	0.84	−0.29	9.13	0	2.31×10^{-4}	−1.08
AUC_{0-inf}	0.18	−0.84	−0.12	−0.48	0	0	−0.41
AUC_{tEnd}	−0.17	−0.86	−0.11	−0.59	0	0	−0.40
C_{max}	-8.28×10^{-4}	−0.69	−0.02	−2.11	0	-5.56×10^{-4}	−1.25
C_{tEnd}	−0.83	−0.22	−0.34	3.89	0	0	−1.25
Half life	−0.20	0.22	−0.07	1.30	0	1.66×10^{-4}	0.24
MRT	−0.24	0.17	−0.09	1.78	0	0	−0.32
t_{max}	0	0	0	0	0	0	0
Cl/F	0.18	0.84	0.12	0.48	0	0	0.41
Vd/F	−0.01	1.07	0.05	1.87	0	2.2×10^{-4}	0.17
Vss/F	−0.06	1.01	0.03	2.36	0	0	0.10

$AUC_{tlast-\infty}$: area under concentration-time curve from last measurable concentration to infinity, AUC_{0-inf}: area under the concentration-time curve from time 0 to infinity, AUC_{tEnd}: area under the concentration-time curve from time 0 to the end of dosing interval, C_{max}: maximum plasma concentration, C_{tEnd}: plasma drug concentration at the end of the dosing interval, MRT: mean residence time, t_{max}: time at maximum plasma concentration, Cl/F: total body clearance/oral bioavailability, Vd/F: volume of distribution/oral bioavailability, Vss/F: volume of distribution at steady state/oral bioavailability, F_u: fraction unbound, K_{cat}: turnover number.

Supplementary S1. Calculation of Mean Gastric Emptying Time (GET) in Infants

1. T½ for liquid GET ($GET_{½}$) (min) in infants

The liquid $GET_{½}$, across two age brackets, was extracted from a scintigraphy study for the target cohort age, i.e., 1–5.5 months.

29 days–3months = 62.67
4–6 months = 69.84

Since the study infants were aged 1–5.5 months, an unweighted simple average of overlapping age brackets was calculated to yield a single composite value of $GET_{½}$, as this method provides an unbiased midpoint.

Mean $GET_{½}$ = 62.67 + 69.84 /2 = 132.51/ 2= 66.255

2. Conversion of $GET_{½}$ to mean GET

The mean $GET_{½}$ was converted to mean GET using the standard first-order kinetic formula, and the calculated value was parameterized in PK-Sim for developing infants' PBPK model

$$\text{Mean GET} = \frac{T_{1/2}}{\ln(2)}$$

Mean GET= 66.26/0.693= 95.6
